# Continuously tunable broadband adiabatic coupler for programmable photonic processors

**DOI:** 10.1515/nanoph-2025-0402

**Published:** 2025-11-06

**Authors:** Xiang Liu, Peipeng Xu, Yingxuan Zhao, Zhen Sheng, Fuwan Gan

**Affiliations:** Shanghai Institute of Microsystems and Information Technology, Chinese Academy of Sciences, No. 865, Changning Road, Shanghai, 200050, China

**Keywords:** silicon photonics, programmable photonic integrated circuits, adiabatic coupler

## Abstract

Programmable integrated photonic circuits are poised to drive a new revolution in information systems by synergizing with high-speed digital signals. Central to this vision is the ability to reconfigure optical signal processing for multi-functional photonic integration. Here, we design and experimentally demonstrate a thermo-optically reconfigurable adiabatic coupler monolithically integrated on a silicon photonics platform. The device combines adiabatic directional couplers with titanium nitride (TiN) micro-heaters embedded in the adiabatic transition region, enabling dynamic coupling ratio tuning via the localized thermo-optic modulation. Experimental results confirm continuous coupling ratio adjustment from 50:50 to 70:30 across 80-nm bandwidth (1,520–1,600 nm), with insertion loss kept below 0.25 dB. Leveraging its tunability, the device enables programmable spectral routing with free spectral ranges (FSR) of 20 nm and 40 nm. The proposed approach offers enhanced flexibility and scalability for high-density photonic systems, providing a promising pathway toward next-generation programmable photonic circuits and optical computing architectures.

## 1 Introduction

Programmable photonic processors have recently emerged as a central platform for advancing optical computing, enabling large-scale reconfigurable transformations that extend far beyond the capabilities of fixed-function photonic circuits [1], [2], [3]. Among the most prominent paradigms are mesh-based architectures, including Mach–Zehnder interferometer (MZI) meshes [4], [5], micro-ring resonators (MRRs) [6], [7] and programmable waveguide networks [8], which allow the implementation of arbitrary unitary operations with high flexibility. Together with other passive components, they serve as fundamental blocks for building more intricate photonic circuits within the core of a comprehensive optical system. Nevertheless, these approaches encounter critical scalability challenges, such as footprint constraints, accumulated crosstalk, and the increasing complexity of thermal or electro-optic tuning [9]. Within these architectures, beam splitters and combiners function as indispensable building blocks, governing signal division, interference, and routing across the optical network [10], [11], [12]. They play a crucial role in adding functionality and enabling the reconfigurable photonic integrated circuits (PICs). Motivated by these considerations, the present work introduces a high-efficient coupler design that can be seamlessly integrated into programmable photonic processors. Conventional power splitters are mainly based on two strategies: mode coupling and mode evolution [13], [14]. Mode-coupling devices offer compact footprints but typically suffer from limited bandwidth and sensitivity to fabrication imperfections and temperature variations [15], [16]. Subwavelength grating (SWG) structures have been employed to engineer the effective index and thereby shorten the coupling length. This approach overcomes the bandwidth limitations but still lacks efficient reconfigurable pathways [17], [18], [19]. Moreover, cascaded couplers have been demonstrated as a practical route toward broadband tunable operation, offering a versatile design concept that could unlock significant potential for scalable photonic integration [20]. Inverse design have recently gained popularity for on-chip optical manipulation [21], [22], enabling compact devices like Y-junctions [23], MMI couplers [24], [25]. This approach enables topology-optimized couplers with ultracompact footprints and low loss. Nevertheless, the resulting complex waveguide patterns present nontrivial fabrication challenges and low reconfigurability. For broader bandwidth and greater reconfigurability, mode-evolution-based couplers gain a great popularity. However, they typically occupy large device footprints, posing a significant challenge for programmable photonic integration [26], [27]. Through three-dimensional femtosecond laser focal field engineering, solutions based on lithium niobate photonic crystals offer great tunability, but are often limited by narrow operational bandwidths and complex fabrication [28]. Alternatively, chalcogenide phase-change materials (PCMs), such as Sb_2_Se_3_, provide large optical contrast and nonvolatile switching capabilities [29]. However, PCM-based devices are often constrained by limited reconfigurable states and are difficult to integrate with CMOS platforms. Current coupler implementations, however, remain constrained by several critical challenges, including fabrication-induced imperfections, fluctuations in operating conditions, and limited reconfigurability. By addressing the trade-offs between coupling precision, footprint, and dynamical control, our approach aims to advance the functionality and stability of next-generation photonic processing systems.

Here, we propose and experimentally demonstrate a CMOS-compatible, tunable broadband power splitter on the SOI platform. The device is based on adiabatic directional couplers integrated with TiN micro-heaters. By tuning the effective refractive index in the adiabatic transition region through the thermo-optic effect, wide-range reconfiguration of the power splitting ratios (PSRs) is achieved. Experimental results show robust control of splitting ratios – 50 %:50 %, 60 %:40 %, and 70 %:30 % – under applied voltages of 0 V, 4.7 V, and 6.6 V within the wavelength range from 1,520 nm to 1,600 nm. With excellent power splitting uniformity and insertion loss (IL) below 0.25 dB, the device significantly enhances the functional versatility of on-chip power splitters and paves the way for reconfigurable, programmable silicon photonic systems. Through tuning couplers and phase shifters at runtime, we can realize programmable multi-purpose filters with 20 nm and 40 nm FSRs, featuring both flatness and low crosstalk.

## 2 Device design and operation principles

**Figure 1:**
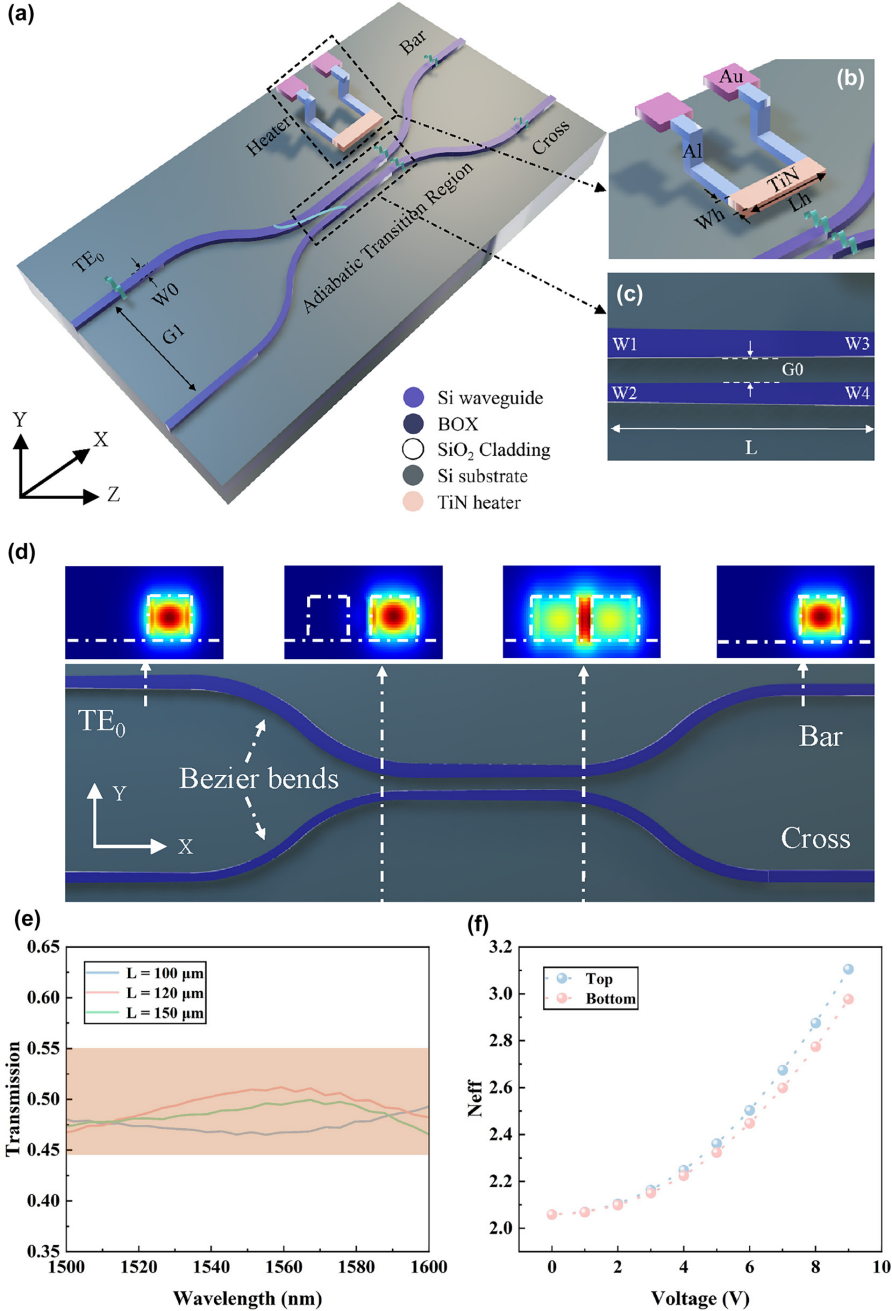
Device structure and operating principle of the reconfigurable adiabatic directional coupler. (a) Schematic diagram of the reconfigurable adiabatic directional coupler integrated with TiN micro-heaters. (b) Schematic of the TiN micro-heater structure. (c) Structural parameters of the adiabatic transition region in the coupler. (d) TE_0_ optical eigenmodes at different positions of the adiabatic directional coupler, corresponding to the input port, the start of the adiabatic transition region, the end of the adiabatic transition region, and the output port, respectively. (e) Power-splitting characteristics of the coupler under top-port excitation and zero bias, for adiabatic lengths of 100 μm, 120 μm, and 150 μm over the wavelength range of 1,500–1,600 nm. (f) Simulated effective refractive index variations of the top and bottom waveguides under 0–9 V applied voltages at 1,550 nm.

**Figure 2:**
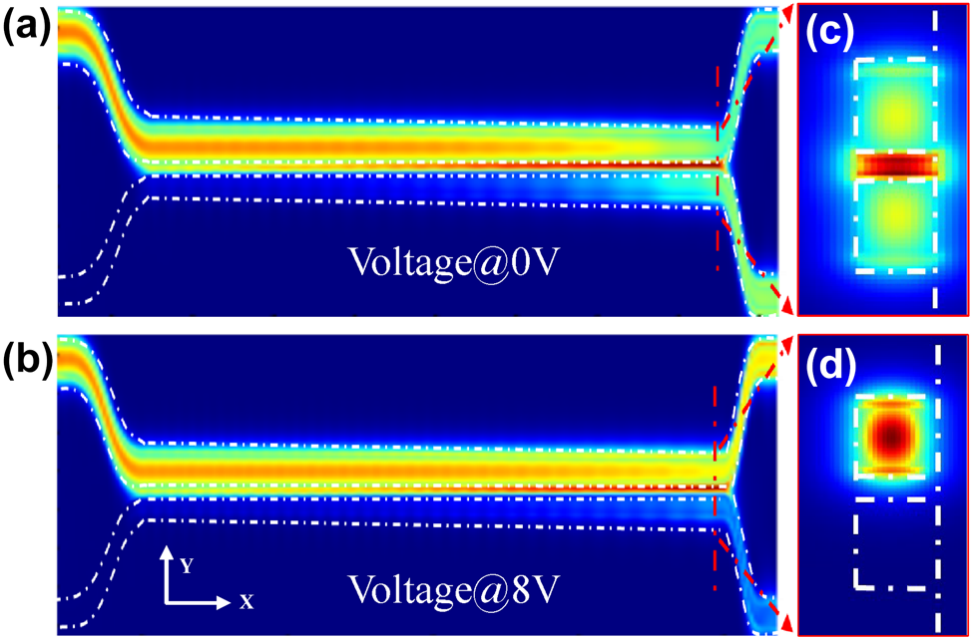
Simulated optical field and mode field distributions under different bias conditions. (a) Simulated optical field profile in the adiabatic coupler under a bias of 0 V. (b) Simulated optical field profile under an applied voltage of 8 V. (c) Optical eigenmodes at the output of the adiabatic transition region under 0 V applied voltage. (d) Optical eigenmodes at the output of the adiabatic transition region under 8 V applied voltage.

According to coupled-mode theory (CMT), the incident fundamental transverse-electric (TE_0_) mode excites both odd and even modes within the coupling region of a directional coupler. These modes undergo periodic coupling, leading to optical power oscillation between the two waveguides. Owing to waveguide dispersion, the PSRs of directional couplers are highly sensitive to wavelength variations. In contrast to conventional directional couplers, adiabatic structures suppress the excitation of odd and even modes by gradually varying the width of the strip waveguides along the propagation direction. Following the mode evolution principle, the incident mode avoids unintended coupling, preserving its form throughout the adiabatic coupler. As a result, the input mode remains stable at the outputs, with only quasi-static evolution along the device. By engineering a graded effective refractive index profile, the structure enables low-loss power splitting between the two output ports. This design greatly improves wavelength insensitivity while ensuring low insertion loss and high fabrication tolerance. Figure 1(a) illustrates the proposed device, built on a standard SOI platform with adiabatic directional couplers. To enable wide-range reconfigurable PSRs, a TiN-based micro-heater is integrated into the adiabatic transition region, as shown in Figure 1(b). This region consists of two opposing isosceles trapezoidal waveguides with an initial gap of G0, as illustrated in Figure 1(c). To guarantee stable single-mode propagation, the width *W*_0_ of the input waveguide is defined as 450 nm with a TE_0_ incident mode. Bezier-shaped waveguides are employed to connect the input/output ports and the mode evolution region, and thereby further reduce the optical loss [30]. Following the mode-evolution principle, effective mode evolution can be attained provided that the taper length is sufficiently long to maintain adiabatic conditions [31]. Besides, sustaining single-mode conditions is crucial to prevent the conversion of the fundamental mode to higher-order mode. At the output ends, where the two adiabatic waveguides have equal widths (*W*_3_ = *W*_4_), the optical signal is evenly split into two beams, as demonstrated in Figure 1(d). By modulating the refractive index distribution through localized heating, the optical power distribution can be dynamically reconfigured. A smaller gap between the waveguides can enhance coupling strength, enabling a more compact adiabatic structure [32]. To balance fabrication tolerance and device performance, the waveguide gap is set to 100 nm, and we evaluated power splitting uniformity for adiabatic lengths of 100 µm, 120 µm, and 150 µm without applied voltages. As shown in Figure 1(e), the device achieves excellent 0.5 ± 0.05 power splitting within the wavelength range from 1,500 nm to 1,600 nm at 0 V. Taking advantage of silicon’s high thermo-optic coefficient (∼1.8 × 10^−4^/K) [33], the TiN heater can modulate the effective refractive index to efficiently tune the PSRs. Figure 1(f) shows the dependence of the effective refractive index tuning on the applied voltages at 1,550 nm. It illustrates the simulated effective refractive index variations of the top and bottom waveguides under different voltages. The bottom waveguide, being closer to the heater, exhibits a larger refractive index change, and the index contrast between the two waveguides increases with the applied power. This trend agrees well with theoretical expectations and provides a physical basis for achieving dynamically tunable power-splitting ratios. Based on a tradeoff between tuning efficiency, power-splitting uniformity and operational bandwidth, the optimized structure employs *L* of 100 µm and *W*_3_ of 350 nm. Figure 2(a) and (b) show the optical field profiles of our proposed device at 0 V and 8 V applied voltage, respectively.

Under different bias, optical field profiles exhibit a significant redistribution due to thermo-optic modulation. These calculated results establish a feasible foundation for dynamical reconfiguration of light splitting. The calculated mode distributions at the end of the adiabatic transition region are presented in Figure 2(c) and (d). According to the mode evolution theory [27], the dependence between the transmission of the bar port and cross port can be expressed as:

(1) $$\frac{T_{\text{bar}}}{T_{\text{cross}}}=1+\frac{2}{\text{tan}{\left(\xi \right)}^{2}}+\frac{2\sqrt{1+\text{ta}{\text{n}}^{2}\left(\xi \right)}}{tan^{2}\left(\xi \right)}$$

(2) $$\tan\left(\xi \right)=\frac{c_{i,i+1}}{\varphi }$$

(3) $$\varphi =\frac{1}{2\left(\beta_{\text{up}}-\beta_{\text{down}}\right)}$$

where *T*_bar_ and *T*_cross_ represent the transmission of the bar port and cross port, respectively; *C*_*i*,*i*+1_ is the coupling coefficient between the *i*-th order mode and *i* + 1-th order mode; *β*_up_ and *β*_down_ are the propagation constants of the upper and lower waveguides, respectively. The PSR can be further defined as follows:

(4) $$\text{PSR}=\frac{T_{\text{bar}}}{T_{\text{bar}}+T_{\text{cross}}}\;\text{or}\;\frac{T_{\text{cross}}}{T_{\text{bar}}+T_{\text{cross}}}$$

**Figure 3:**
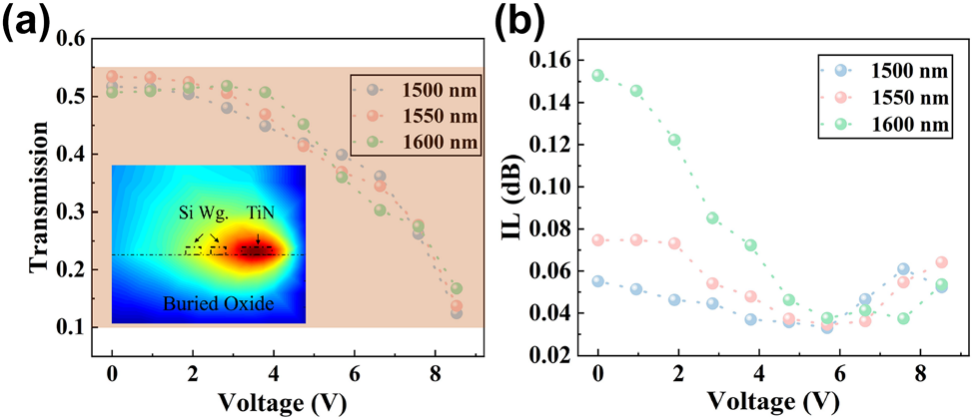
Simulated tuning performance and IL analysis of the tunable adiabatic coupler. (a) Simulated cross-port transmission as a function of applied voltage at wavelengths of 1,500 nm, 1,550 nm, and 1,600 nm. The inset image shows the simulated thermal distribution generated by the TiN microheater with a bias of 5 V. (b) Corresponding simulated IL for the device across the full tuning range and operational bandwidth.

**Figure 4:**
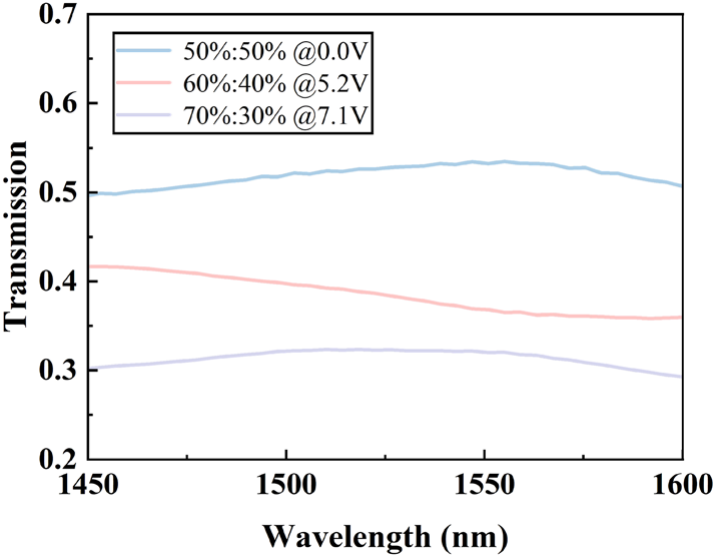
Simulated cross-port transmission of the adiabatic coupler under top-port excitation over the 1,450–1,600 nm wavelength range, for applied voltages of 0 V, 5.2 V, and 7.1 V.

Based on the above analysis and simulation results, the proposed device enables wide-range reconfiguration of the PSRs at the wavelengths of 1,500 nm, 1,550 nm, and 1,600 nm by varying the applied voltage from 0 to 9 V. As shown in Figure 3(a), the splitting ratios can be reconfigured from 10 %:90 % to 50 %:50 %, offering a wide-range programmability. We also evaluated the IL under different applied voltages, and as shown in Figure 3(b), the IL remains below 0.16 dB at the wavelengths of 1,500 nm, 1,550 nm, and 1,600 nm. This device breaks through the limitations that conventional silicon-based splitters achieve arbitrary splitting ratios (ASRs) through changing structural parameters and can be only employed in application-specific PICs. We calculated the beam-splitting performance within the optical communication band under different bias, as shown in Figure 4. Calculated results demonstrate that the proposed device offers excellent broadband operation across the wavelength range of 1,450–1,600 nm at 0 V, 5.2 V, and 7.1 V. By elaborately combining mode evolution with the thermo-optic effect, we realize a CMOS-compatible 2 × 2 power splitter with a 150 nm operating bandwidth and excellent dynamic tunability. These features enable flexible switching among different PSRs, offering a promising solution for applications in programmable photonic systems.

## 3 Device characterization and analysis

**Figure 5:**
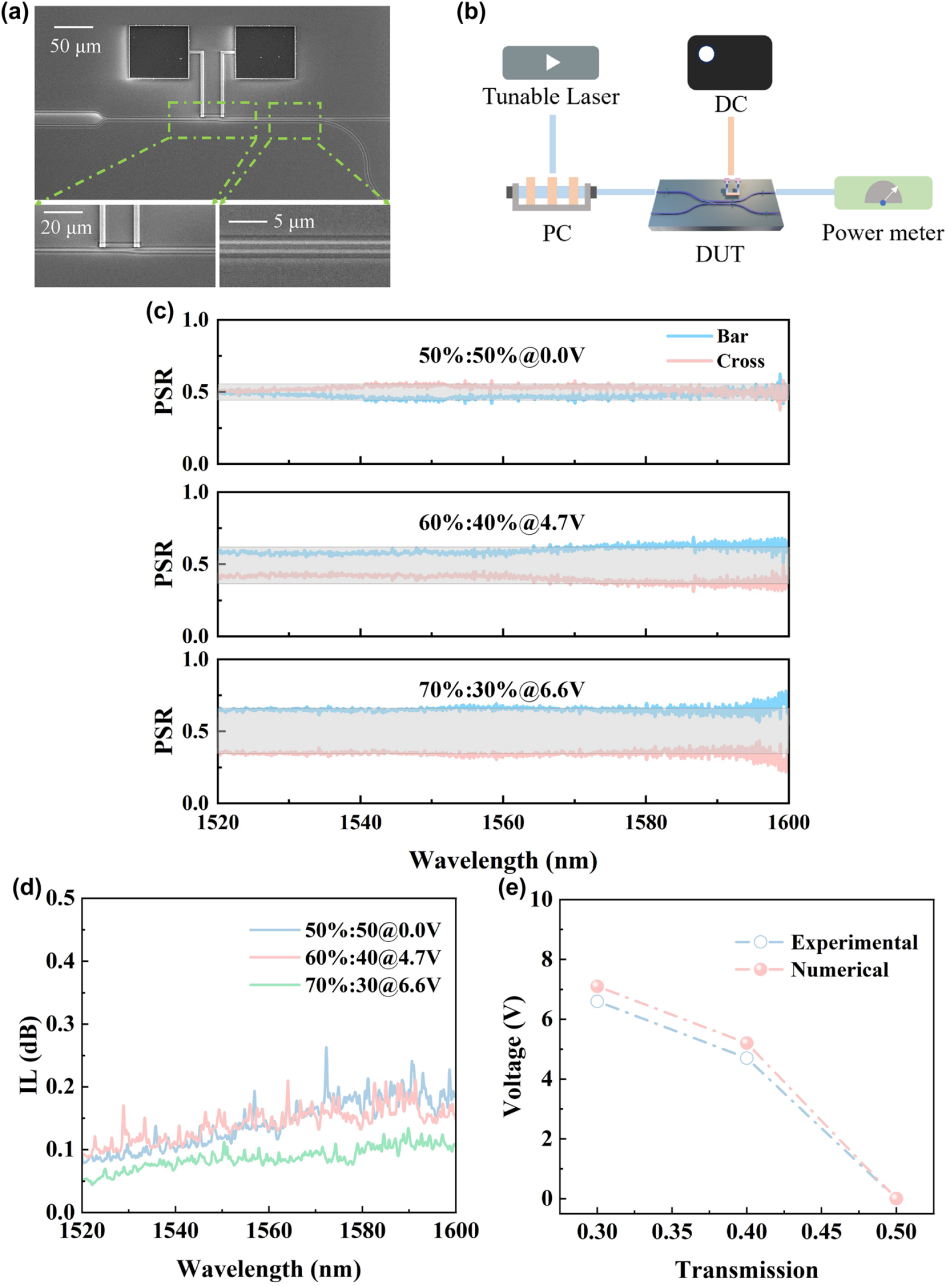
Experimental demonstration and characterization of the reconfigurable adiabatic power splitter. (a) SEM image of the fabricated device, highlighting the central coupling region. (b) Schematic of the experimental setup used for the optoelectronic characterization, including a tunable laser source, polarization controller, DC voltage source and optical power meters. (c) Measured PSRs under different applied voltage in the wavelength range of 1,520 nm–1,600 nm, showing continuous tuning across the C- and L-bands. (d) Measured IL of the coupler under different bias conditions after subtracting the calibrated fiber-to-chip coupling loss. For all measurements in (c) and (d), the device was excited via the top input port. (e) Comparison of the measured (blue line) and simulated (red line) bias as a function of transmission at a representative wavelength of 1,550 nm.

**Figure 6:**
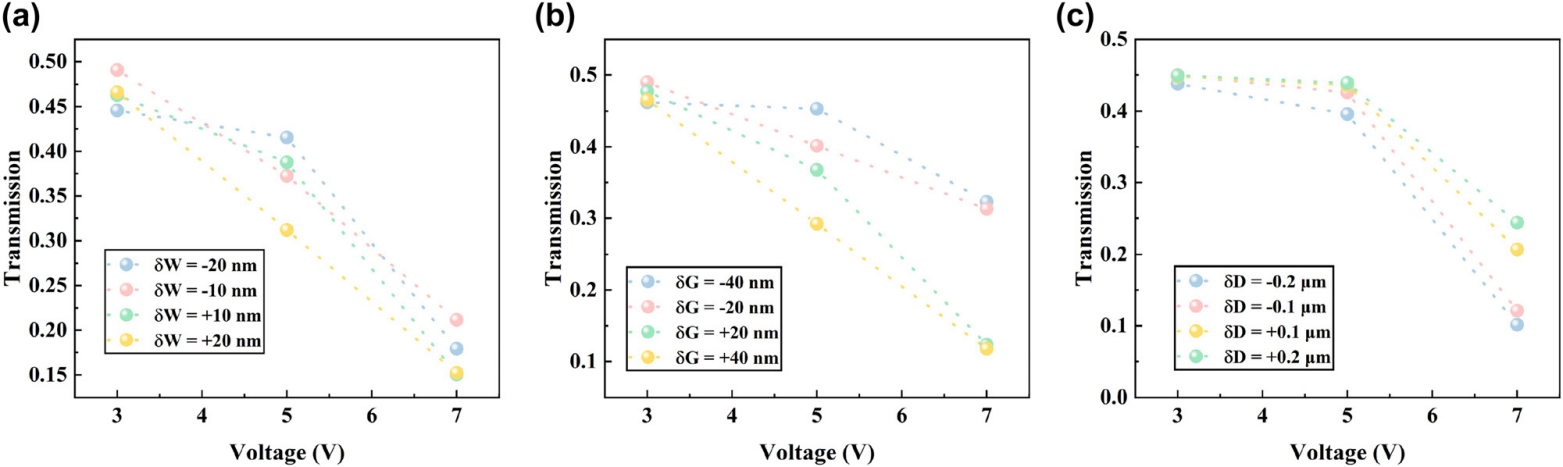
Simulated robustness of the device against fabrication non-idealities. The device’s tuning characteristics are simulated at a wavelength of 1,550 nm to assess the impact of key geometric variations from the nominal design. (a) Transmission response for variations in the waveguide core width (δW) of up to ±20 nm. (b) Corresponding response for variations in the inter-waveguide coupling gap (δG) of up to ±40 nm. (c) The effect of a ±0.2 µm deviation in the distance (δD) between the microheater and the waveguide.

The device was fabricated on standard SOI wafers, consisting of a 220 nm silicon core layer, a 2 μm buried oxide layer, and a 500 μm silicon substrate. High-resolution electron-beam lithography (EBL) was employed to define the device patterns onto the top silicon layer in ZEP-520A resist, followed by reactive ion etching (RIE) to form the waveguide structures. TiN thin film was deposited via ion-beam sputtering, and then the TiN micro-heater was formed onto the silicon core layer through inductively coupled plasma (ICP) dry etching. Aluminum (Al) was deposited as metal interconnects near the TiN heaters using ion-beam sputtering. A 2 μm-thick silicon dioxide layer was then deposited as a passivation layer, with openings etched to form Au contact pads. Scanning electron microscope (SEM) images of the fabricated device are shown in Figure 5(a), demonstrating excellent consistency between the experimental structure and the design. To characterize the device performance, the test setup illustrated in Figure 5(b) was established. A broadband tunable laser (Santec TSL-550, 1,500–1,630 nm) served as the light source, with light coupled through a single-mode fiber. A polarization controller (PC) was used to align the polarization state of the incident light. The TiN micro-heater was driven by a DC voltage source (Tektronix 2280S), and the output power under different bias was recorded using a multi-channel power-meter (Jointwit JW8103D). In experimental characterization, the top port was used as the primary input port, as the device is optimized for this excitation condition. Experimental results show that with zero bias, the device achieves a 50 %:50 % PSR within 0.5 ± 0.05 over the wavelength range of 1,520–1,600 nm. By applying voltages of 4.7 V and 6.6 V, the PSRs can be reconfigured to 60 %:40 % and 70 %:30 % at runtime, as shown in Figure 5(c). The measured results validate the device’s excellent tunability, confirming the feasibility of dynamically reconfiguring the PSRs through the combination of adiabatic mode evolution and thermo-optic tuning. As shown in Figure 5(d), the IL remains below 0.25 dB across different bias conditions. The reported values have been calibrated by subtracting the fiber-to-chip coupling loss using a reference straight waveguide fabricated adjacent to the device under test. The observed IL is slightly higher than the calculated results, possibly attributed to the rougher sidewalls of the fabricated silicon waveguides, which lead to increased scattering loss. The measured PSRs at different bias exhibit certain deviations from the calculated results, as depicted in Figure 5(e). The modest discrepancy observed between the measured and simulated splitting ratios can be attributed to unavoidable fabrication non-idealities. The most critical of these are geometric deviations from the nominal design, including variations in the waveguide core width and the inter-waveguide gap, as well as the waveguide-heater gap. To systematically quantify the influence of these parameters, we performed a comprehensive tolerance analysis, with the results presented in Figure 6(a)–(c). Our simulations reveal that variations in the waveguide width, coupling gap and waveguide-heater gap indeed introduce performance shifts. Collectively, these results confirm that the observed deviations fall within the expected range attributable to standard fabrication tolerances, thereby validating our device model. As shown in Table 1, we compared the performance of our work with the state-of-the-art arbitrary-ratio power splitters, including reconfigurability, operational bandwidth, insertion loss, footprint and power consumption. Conventional methods through changing the physical structure can achieve tunable PSRs prior to device fabrication [23], [27], [29]. However, such devices are only well-suited for the application-specific PICs. Once the devices are integrated on-chip, their functionality cannot be further modified. In contrast to such devices, our approach enables superior reconfigurability via thermo-optic tuning based on a fixed device structure, thereby significantly unlocking the application potential for programmable PICs. In addition, chalcogenide PCMs offer high optical contrast between their crystalline and amorphous states, making them a suitable choice for reconfigurable devices [34], [35], [36], [37]. However, PCM-based power splitters typically can only switch between limited discrete states. Moreover, the complex fabrication process of PCM-based splitters is not compatible with CMOS technology.

**Table 1:** Performance comparison of this work with the state-of-the-art power splitters enabling arbitrary splitting ratios.

Reference	Structure	Footprint (μm)	IL (dB)	Bandwidth (nm)	Reconfigurability ranges (dB)	Power efficiency (mW)	Evaluation methods
[38]	Slotted ring resonator	200	NA	NA	0.03–16.7	NA	Experimental
[27]	Adiabatic coupler	240	NA	100	N	NA	Experimental
[39]	Rapid adiabatic couplers	31	<0.5	145	N	NA	Experimental
[40]	SWG-based MMI	116.5	1.5	40	0.4–35	27	Experimental
[34]	Sb_2_Se_3_-assisted MMI	33	0.5	NA	0–10.61	0	Experimental
[35]	GSST-based MMI	3.6	NA	30	0–3.98	0	Numerical
[36]	Sb-based MMI	1.8	NA	20	0–9.54	0	Numerical
[37]	Sb_2_Se_3_ assisted directional coupler	26.6	0.11	NA	−19.9–15.1	0	Numerical
[23]	Inverse designed Y-junction	14	0.54	120	N	N	Experimental
[41]	Inverse designed MMI	1.96	0.38	50	N	N	Numerical
[42]	Rapid adiabatic couplers	<70.8 µm	NA	>119	N	NA	Experimental
[43]	Adiabatic coupler	1.44 µm	0.03	120	N	NA	Numerical
This work	Tunable adiabatic coupler	100	0.25	80	0–9.54	25.1	Experimental

## 4 Programmable multi-stage filter design

**Figure 7:**
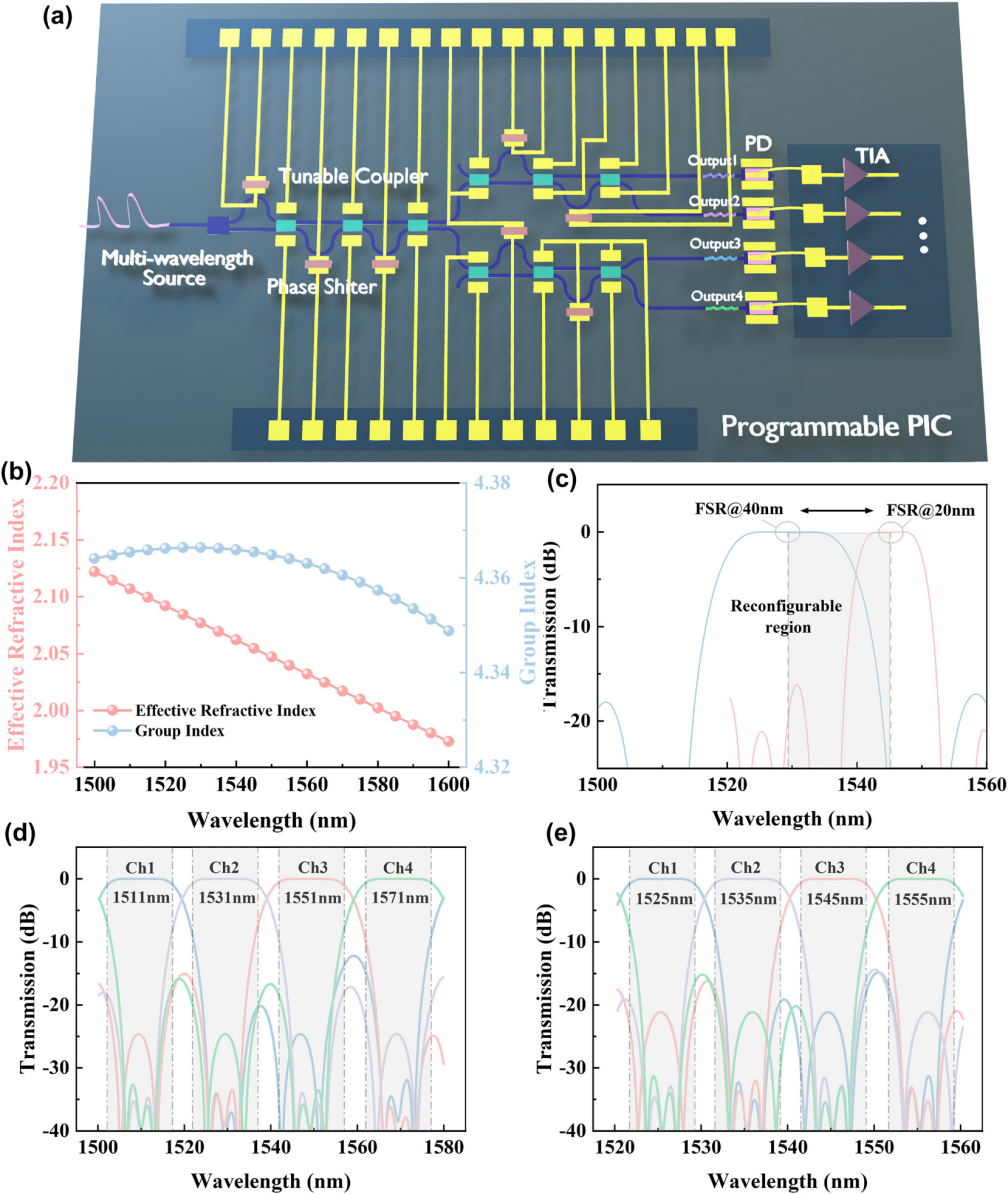
Programmable PIC enabling WDM reconfiguration. (a) Schematic of the programmable PICs for multi-purpose WDM. (b) Distributions of effective refractive index and group index of the silicon waveguide of 450 nm width. (c) Reconfiguration of the multiplexer’s FSR from 40 nm to 20 nm via PSO algorithm. (d) Spectral response of the WDM with a 40 nm FSR in the initial state. (e) Spectral response of the reconfigured multiplexer with a 20 nm FSR.

Our proposed device can achieve a broadband tuning of PSRs for continuous operation, enabling it to meet the demands of complex optical communication systems. Figure 7(a) illustrates the designed programmable multi-stage filter, which implements multi-functional wavelength-division (de)multiplexing (WDM) based on the proposed tunable power splitters and phase shifters. Figure 7(b) presents the effective refractive index and group index of the silicon waveguide, which guide the theoretical tuning parameters of various components. By adjusting the amplitude of the electrical signal, the coupling coefficients and delay lengths are tuned to reconfigure the FSR and central wavelengths. As shown in Figure 7(c), the FSR is reconfigured from 40 nm to 20 nm, accompanied by a central wavelength shift from 1,531 nm to 1,545 nm. Fine-tuning the programmable components further enables adaptive control of the 1 dB bandwidth and channel crosstalk. Figure 7(d) shows the initial state of the programmable multiplexer, featuring an FSR of 40 nm with central wavelengths at 1,511 nm, 1,531 nm, 1,551 nm, and 1,571 nm. Using the particle swarm optimization (PSO) algorithm, the coupling coefficients of the first-stage filter are determined as K1 = 0.5, K2 = 0.15, K3 = 0.24, and K4 = 0.02, while the delay lengths are set to L1 = 28.9 μm, L2 = 57.9 μm, and L3 = 58.1 μm. For the second-stage filter, the coupling coefficients are set to K5 = 0.5, K6 = 0.26, and K7 = 0.08, with corresponding delay lengths of L4 = 13.9 μm, L5 = 27.8 μm, L6 = 14.2 μm, and L7 = 28.5 μm. With these configurations, the WDM achieves a 1 dB bandwidth of 14.8 nm and an average channel crosstalk below −25.2 dB. To verify the programmability of the multiplexer, the coupling coefficients of the first-stage filter are subsequently tuned to 0.5, 0.18, 0.21, and 0.02, with phase adjustments of 0.2*π*, 0.4*π*, and 0.4*π*. The second-stage filter’s coupling coefficients are adjusted to 0.5, 0.29, and 0.09, with the phase adjustment amounts set to 1.4*π*, 0.8*π*, 0.7*π* and 1.7*π*. By programming the electrical signal amplitude, the FSR is dynamically tuned to 20 nm, and the central wavelengths are adjusted to 1,525 nm, 1,535 nm, 1,545 nm, and 1,555 nm. As shown in Figure 7(e), the reconfigured multiplexer achieves a 1 dB bandwidth of 7.6 nm and maintains an average channel crosstalk below −22.8 dB, demonstrating the system’s capability to implement multiple filtering standards. Additionally, the programmable multiplexer offers flexibility beyond WDM functions. Algorithmic fine-tuning enables band switching between the C-band and O-band. The broadband and fabrication-tolerant response of the proposed thermo-optic couplers, together with their efficient and continuously tunable nature, suppresses crosstalk accumulation in large meshes through closed-loop calibration. Power consumption and thermal effects are alleviated by minimal tuning overhead, optimized chip layout, and localized thermal engineering that enhances efficiency and lowers per-element power. Compared with phase-change or doping-based alternatives, our fully CMOS-compatible approach avoids excess loss and process complexity, providing a practical and scalable route to large-scale programmable photonic processors. This dynamically reconfigurable device effectively compensates for PSR deviations in conventional splitters, offering a versatile solution for programmable photonic circuits and real-time optical control in high-performance computing.

## 5 Conclusions

In this work, we present and experimentally demonstrate a continuously tunable adiabatic coupler for monolithic programmable PICs. Conventional silicon-based splitters are often sensitive to fabrication imperfections and lack effective tuning mechanisms. By leveraging mode-evolution principles and integrating TiN-based micro-heaters, we realize a dynamically tunable architecture that overcomes these limitations. Experimental results demonstrate that our proposed device can realize the PSRs reconfiguration of 50 %:50 %, 60 %:40 %, and 70 %:30 % under applied voltages of 0 V, 4.7 V, and 6.6 V, with excellent splitting uniformity over an 80 nm bandwidth and insertion loss below 0.25 dB at runtime. Through tuning couplers and phase shifters at runtime, the programmable multi-purpose filters with 20 nm and 40 nm FSRs are proposed, featuring both flat spectral response and low crosstalk. With its broad tuning range and low loss, the proposed device offers a flexible solution for programmable PICs, paving the way for next-generation applications in optical communication, sensing, and on-chip computing.
